# Hyperkalaemic acidosis: blood pressure is the diagnostic clue

**DOI:** 10.1007/s00467-024-06590-4

**Published:** 2024-11-11

**Authors:** Jakub Zieg, Dana Thomasová, Malgorzata Libik, Zdenek Sumnik, Detlef Bockenhauer

**Affiliations:** 1 Department of Pediatrics, Second Faculty of Medicine, Charles University and Motol University Hospital, Prague, Czech Republic; 2 Institute of Biology and Medical Genetics, Second Faculty of Medicine, Charles University and Motol University Hospital, Prague, Czech Republic; 3 Department of Pediatric Nephrology, University Hospital and Catholic University Leuven, Herestraat 49, 3000 Leuven, Belgium; 4 Department for Renal Medicine, University College London and Great Ormond Street Hospital for Children, London, UK

**Keywords:** Gordon syndrome, Hyporeninemic hypertension, Pseudohypoaldosteronism type 2, Thiazides

## Abstract

Pseudohypoaldosteronism type 2 (PHA2) is a rare inherited condition of altered tubular salt handling. It is characterized by the specific constellation of hyperkalaemic hyporeninemic hypertension, hyperchloremic metabolic acidosis and hypercalciuria. Molecular genetic testing confirms the diagnosis in the majority of cases. Thiazides constitute effective treatment. Due to its rarity, the diagnosis is often delayed. We here present two children with PHA2, who were initially treated with fludrocortisone and bicarbonate complicated mainly by exacerbation of their hypertension. Discontinuation of their previous therapy and commencement of thiazide diuretics led to normalisation of their blood pressure and electrolyte and acid–base status.

## Background

Pseudohypoaldosteronism type 2 (PHA2), also known as Gordon syndrome, is a rare genetic disorder associated with variants in *WNK1*, *WNK4*, *KLHL3*, and *CUL3* [1]. It is typically inherited in an autosomal dominant manner except that *KLHL3* can also be associated with recessive disease. All 4 disease genes encode proteins directly or indirectly involved in regulation of the sodium chloride co-transporter in the distal convoluted tubule (DCT) with disease-causing variants causing over-activity of the transporter with enhanced sodium and chloride reabsorption. The consequent impaired delivery of sodium to the collecting duct results in impaired aldosterone-mediated exchange of sodium for potassium and protons, resulting in hyperkalaemia and hyperchloremic metabolic acidosis, despite normal aldosterone production (pseudohypoaldosteronism). Yet, as the primary problem is enhanced sodium reabsorption, PHA2 is associated with hypertension, which distinguishes it from aldosterone deficiency and PHA type 1, where the primary problem is impaired sodium reabsorption with consequent hypotension. Thus, blood pressure assessment is a key diagnostic clue, and here, we present 2 cases that highlight its importance for correct diagnosis and treatment.

## Case 1

A 3-year-old girl was admitted due to an incidental finding of hyperkalaemia and metabolic acidosis (Table 1) at the pre-operative work-up for a minor orthopaedic procedure. Her family history was unremarkable apart from hypertension in a maternal aunt treated with two antihypertensive drugs. The girl was born to non-consanguineous parents following a pregnancy complicated by intrauterine growth retardation suspected by ultrasonography at the 28th week of gestation. She was delivered at term; her birth weight and length were 2260 g and 45 cm, respectively. On admission, her height was 90.2 cm (3rd percentile) and BMI 15 kg/m^2^ (25th percentile). Her blood pressure was 119/60 (systolic > 95th percentile, diastolic 90th percentile). Her physical examination was normal. Her kidney sonography showed normal finding. Transtubular potassium gradient was 1, which indicated aldosterone deficiency or resistance. Repeated blood pressure measurements showed hypertension stage 1 with a systolic BP range of 119–127 mmHg. Despite this, a clinical diagnosis of hyporeninemic hypoaldosteronism was made, and treatment with fludrocortisone 50 mcg twice daily and sodium bicarbonate 6 mmol/kg/day was commenced with subsequent normalisation of serum potassium and bicarbonate. Her hypertension was treated with amlodipine at the dose of 0.2 mg/kg/day. Molecular genetic testing was not available at that time. She was followed-up by a paediatric endocrinologist due to short stature (− 2.0 SD) and growth retardation that developed after the age of 8 years with a minimum height SD of − 2.8 SD. She reached a final height of 160 cm, which corresponds to the parental heights. Upon review of her clinical phenotype at the age of 15 years, the question of a potential diagnosis of PHA2 was raised and genetic testing was done, which identified two variants in *KLHL3* (NM 017415.3) classified as class 4 (likely pathogenic) according to the American College of Medical Genetics and Genomics guidelines (ACMG.net): c. 1229C > T (PM1, PM2, PP3, PP5) and c.827A > G (PM2, PP3, PS2). The *KLHL3* c.1229C > T p.(Ser410Leu) variant was previously described as disrupting the interaction of *KLHL3* with *WNK1*, which via accumulation of *WNK1* impairs down-regulation of NCC activity [2, 3]. This variant was inherited from the mother. The other variant c.827A > G p.(His276Arg) is a missense variant affecting a highly conserved nucleotide. This variant with a very low allele frequency of 0.00000124 in gnomAD (https://gnomad.broadinstitute.org/) was found to be “de novo”. Treatment was switched from fludrocortisone with bicarbonate to hydrochlorothiazide therapy (0.5 mg/kg/day) with resolution of her hypertension and normal electrolyte and acid–base status. Moreover, her urinary calcium excretion also normalised (Table 1).

**Table 1:** Pertinent results

	Case 1			Case 2		
	Presentation	Initial therapy	Thiazide	Presentation	Initial therapy	Thiazide
Age (years)	3	10	15	0.05	0.1	0.15
BP (mmHg)	119/60	134/84	124/77	NA	124/-	84/-
BP percentiles	> 99th/90th	> 99th/ > 99th	90th/90th	NA	> 99th	< 50th
Na (mmol/L)	134	145	135	133	137	134
K (mmol/L)	7.6	4.1	3.9	7.4	6.7	3.2
Cl (mmol/L)	113	105	108	NA	NA	NA
HCO_3_ (mmol/L)	11.3	21.3	25	16	24	26
Creatinine (µmol/L)	46	32	72	40	31	25
eGFR (mL/min/1.73 m^2^)	75	111	80	45	59	70
Albumin (g/L)	46	NA	63	NA	NA	NA
Aldosterone	17 ng/dL (25–130)*	13.5 ng/dL (25–130)*	NA	3480 pmol/L (100–800)*	758 (100–800)*	NA
Plasmatic renin activity/renin^$^	0.268 ng/mL/h (0.8–2.0)*	0.508 ng/mL/h (0.8–2.0)*	NA	< 0.3 pmol/L (> 1)*	NA	NA
Urinary Ca/creatinine (mmoL/mmoL)	2.83 (< 1.5)*	1.00 (< 0.7)*	0.12 (< 0.7)*	NA	NA	NA
**–––––––––––––––––––––––––––––––––––––––––––––––––––––––––––––––––––––––––––**						
Parameters of calcium phosphate metabolism at presentation	Calcium 2.47 mmol/L (2.05–2.54)*	Phosphate 1.87 mmol/L (1.16–1.9)*	PTH 2.47 pmol/L (1.3–7.6)*	NA	NA	NA

Shown are pertinent data regarding blood pressure and laboratory results. Normal ranges are shown in brackets, if applicable. Note the elevated blood pressure on “Initial therapy”, which consisted of fludrocortisone and sodium supplementation, and normalisation of blood pressure and electrolytes on thiazide treatment

*eGFR* estimated glomerular filtration rate, *NA* not available, *PTH* parathormone

^$^In case 1, plasmatic renin activity was measured, whereas it was renin concentration in case 2

*Normal values

## Case 2

A 10-day-old girl was referred by the visiting nurse because of insufficient weight gain. Her birthweight had been 2320 g, and her current weight was 2275 g. Pregnancy had been complicated by IUGR, and she was born at 37-week gestation. Her clinical examination was unremarkable, and the admitting doctor noted good peripheral perfusion with no signs of dehydration. Laboratory investigations showed mild hyponatraemia with hyperkalaemic acidosis (Table 1). A preliminary diagnosis of aldosterone insufficiency was made and treatment with sodium bicarbonate, sodium chloride, sodium-polystyrene sulfonate and fludrocortisone with eventual dosages of 13 mmol/kg/d, 10 mmol/kg/d, 600 mg/kg/d and fludrocortisone 75 mcg/d, respectively. Further investigations included a normal synacthen test and normal urinary steroid profile, and the initial (pre-treatment) aldosterone level was elevated with suppressed renin (Table 1). The girl developed severe hypertension during her treatment, with systolic blood pressure mostly above 100 mmHg (range: 88–136). Treatment with furosemide was commenced, and she was transferred to nephrology for further management. There, PHA2 was suspected, all current treatment was stopped, and treatment with chlorothiazide 20 mg/kg twice daily commenced, resulting in complete normalisation of her blood pressure and electrolytes (Table 1). Genetic testing identified a homozygous splice-site mutation in *KLHL3* (c.903G > A; p. = ; PVS1, PM2, and PP4) confirming the diagnosis.

## Discussion

Our cases provide several clinical insights. First, they highlight the importance of volume assessment in patients with hyperkalaemic acidosis, which is the biochemical “fingerprint” of impaired sodium reabsorption in the collecting duct [4]. Because of the critical importance of sodium for volume homeostasis, a defect in sodium reabsorption is usually expected to cause hypovolaemia and, indeed, aldosterone insufficiency or a defect in its effector molecules; the mineralocorticoid receptor and the epithelial sodium channel ENaC (PHA type 1) are both associated with severe hypovolaemia. In contrast, as detailed above, PHA2 is associated with hypervolemia and high blood pressure. Making the distinction between hypo- and hypervolaemic hyperkalaemic acidosis is therefore critical, as it directly affects treatment. In hypovolaemia, the treatment is mineralocorticoid and/or salt supplementation, whereas this exacerbates the hypervolaemia in PHA2, resulting in deterioration of hypertension, as seen in our patients. Instead, thiazide treatment readily resolves the abnormalities in volume and electrolyte homeostasis, as also seen in our cases. While aldosterone levels in PHA2 can be variable, they are typically low for the degree of hyperkalaemia, due to the hypervolemia with suppressed renin [5]. Second, whereas PHA2 typically manifests later in childhood or even adulthood, our second case shows that at least the recessive form can manifest as early as the neonatal period and thus has to be in the differential diagnosis of hyperkalaemic acidosis also in this age group, if the patient has clinical hypervolaemia. The hypervolemia is likely also the cause for the hypercalciuria, a typical feature of PHA2 and mediated by compensatory altered proximal sodium reabsorption, mirroring the hypocalciuria of Gitelman syndrome [6]. Lastly, we note that in both our patients, mild IUGR was noted. This is not a recognised feature in recessive PHA2, and further cases are needed to confirm this previously unrecognised feature.

## Summary

### What is new?

- PHA2 can occur even in neonates and should be considered in children with hyporeninemic hypertension, hyperkalaemia, metabolic acidosis and normal GFR. Understanding of pathophysiology facilitates proper diagnosis and treatment.

## Data Availability

The data that support the findings of this case are available from the corresponding author.
